# Association Between Children With Life-Threatening Conditions and Their Parents’ and Siblings’ Mental and Physical Health

**DOI:** 10.1001/jamanetworkopen.2021.37250

**Published:** 2021-12-20

**Authors:** Chris Feudtner, Russell T. Nye, Jackelyn Y. Boyden, Katherine E. Schwartz, Emilie R. Korn, Aaron G. Dewitt, Amy T. Waldman, Lisa A. Schwartz, Yuming A. Shen, Michael Manocchia, Rui Xiao, Blyth T. Lord, Douglas L. Hill

**Affiliations:** 1 Justin Michael Ingerman Center for Palliative Care, The Children’s Hospital of Philadelphia, Philadelphia, Pennsylvania; 2 Division of General Pediatrics, Department of Pediatrics, Perelman School of Medicine at the University of Pennsylvania, Philadelphia; 3 Division of Neonatology, Department of Pediatrics, Perelman School of Medicine at the University of Pennsylvania, Philadelphia; 4 Division of Cardiology, Department of Pediatrics, Perelman School of Medicine at the University of Pennsylvania, Philadelphia; 5 Department of Neurology, Perelman School of Medicine at the University of Pennsylvania, Philadelphia; 6 Division of Oncology, Department of Pediatrics, Perelman School of Medicine at the University of Pennsylvania, Philadelphia; 7 Cigna, Bloomfield, Connecticut; 8 Department of Sociology, University of North Florida, Jacksonville; 9 Department of Biostatistics, Epidemiology and Informatics, Perelman School of Medicine at the University of Pennsylvania, Philadelphia; 10 Courageous Parents Network, Newton, Massachusetts

## Abstract

**Importance:**

Despite concerns regarding the potential deleterious physical and mental health outcomes among family members of a child with a life-threatening condition (LTC), few studies have examined empirical measures of health outcomes among these family members.

**Objectives:**

To examine whether mothers, fathers, sisters, and brothers of children with 1 of 4 types of pediatric LTCs have higher rates of health care encounters, diagnoses, and prescriptions compared with families of children without these conditions.

**Design, Setting, and Participants:**

This retrospective cohort study included US families with commercial insurance coverage from a single carrier. Children who had 1 of 4 LTCs (substantial prematurity, critical congenital heart disease, cancer, or a condition resulting in severe neurologic impairment) were identified by a diagnosis in their insurance claim data between July 1, 2015, and June 30, 2016. Each case child and their family was matched with up to 4 control children and their families based on the age of the case and control children. Data were analyzed between August 2020 and March 2021.

**Exposures:**

Having a child or sibling with substantial prematurity, critical congenital heart disease, cancer, or a condition resulting in severe and progressive neurologic impairment.

**Main Outcomes:**

Rates of occurrence of health care encounters, physical and mental health diagnoses, and physical and mental health medication prescriptions, identified from insurance claims data, were compared between case and control families using a multivariable negative binomial regression model. The statistical analysis adjusted for observed differences between case and control families and accounted for clustering at the family level.

**Results:**

The study included 25 528 children (6909 case children [27.1%] and 18 619 control children [72.9%]; median age, 6.0 years [IQR, 1-13 years]; 13 294 [52.1%] male), 43 357 parents (11 586 case parents [26.7%] and 31 771 control parents [73.3%]; mean [SD] age, 40.4 [8.1] years; 22 318 [51.5%] female), and 25 706 siblings (7664 case siblings [29.8%] and 18 042 control siblings [70.2%]; mean [SD] age, 12.1 [6.5] years; 13 114 [51.0%] male). Overall, case mothers had higher rates of the composite outcome of health care encounters, diagnoses, and prescriptions compared with control mothers (incident rate ratio [IRR], 1.61; 95% CI, 1.54-1.68), as did case fathers compared with control fathers (IRR, 1.55; 95% CI, 1.46-1.64). Sisters of children with LTCs had higher rates of the composite outcome compared with sisters of children without LTCs (IRR, 1.68; 95% CI, 1.55-1.82), as did brothers of children with LTCs compared with brothers of children without LTCs (IRR, 1.70; 95% CI, 1.56-1.85).

**Conclusions and Relevance:**

In this cohort study, mothers, fathers, sisters, and brothers who had a child or sibling with 1 of 4 types of LTCs had higher rates of health care encounters, diagnoses, and medication prescriptions compared with families who did not have a child with that condition. The findings suggest that family members of children with LTCs may experience poorer mental and physical health outcomes. Interventions for parents and siblings of children with LTCs that aim to safeguard their mental and physical well-being appear to be warranted.

## Key Points

**Question:**

Do family members of children with a serious pediatric illness have higher rates of health care encounters, diagnoses, and prescriptions?

**Findings:**

In this cohort study of family members of 6909 children with 1 of 4 types of serious pediatric illness and 18 619 control children without illness, mothers, fathers, sisters, and brothers each had higher overall rates of health care encounters, diagnoses, and prescriptions compared with family members of control children.

**Meaning:**

The findings suggest that family members of children with a serious pediatric illness may have increased physical and mental health care needs.

## Teaser

This cohort study examines whether family members of children with 1 of 4 types of life-threatening pediatric conditions have higher rates of health care encounters, diagnoses, and prescriptions compared with families of children without these conditions.

## Introduction

When an infant, a child, or an adolescent has a life-threatening condition (LTC), adverse collateral effects on the mental and physical well-being of parents and siblings have been observed.^1,2,3,4^ Qualitative studies have found that parents of seriously ill children reported negative effects in their own physical health, family life, marriage, social life, finances, education, and career.^1,5^ A qualitative metasynthesis observed that parents of children with LTCs often felt like they were on a “relentless rollercoaster of highs and lows” significantly impacting parents’ emotional well-being and familial relationships.^2^^,p95^ Quantitative studies have found self-reported lower resilience, greater symptoms of posttraumatic distress, greater emotional distress, higher levels of anxiety, more health problems, fewer healthy behaviors, more unhealthy behaviors, sleep disturbances, and lower quality of life among parents of children with LTCs.^6,7,8,9,10,11,12,13,14,15^ If their child is hospitalized, parents may experience high levels of distress both during and after the hospitalization.^9,16,17,18^ Research regarding siblings, with few exceptions,^3,4^ has focused on those whose sister or brother had cancer; these siblings may experience greater stress, depression, anxiety, problems with eating and sleeping, behavioral problems, lower quality of life, disruption of academic and social life, and poorer relationships with their parents.^10,19,20,21,22^

To date, most of these studies have been limited by small sample sizes, self-reported outcomes, single health care institutional settings, a lack of comparator family member outcomes, or a restricted focus on 1 specific form of pediatric LTC (most often, cancer). To address these limitations, we designed this study to use a large commercial health insurance claims data set including insured families located across the US to evaluate the outcomes associated with having a child with 1 of 4 different types of LTCs that have been shown in prior research to be particularly stressful for families: substantial prematurity,^23,24,25^ critical congenital heart disease,^26,27,28^ cancer,^13,29,30^ and conditions resulting in severe and progressive neurologic impairment.^31,32,33^ Families of children with these LTCs were matched to families of similar-aged children without these conditions, and the mental and physical health of parents and siblings were compared as measured by the diagnoses and prescriptions they received. We hypothesized that parents and siblings of a child with 1 of these conditions would be more likely than control family members to receive diagnoses related to mental health, physical health, and physical trauma; to receive prescriptions for mental health and all other conditions; and to experience specific types of health care encounters (hospital admissions, emergency department visits, and outpatient urgent care).

## Methods

This cohort study used deidentified data and did not constitute human participant research; thus, the Children’s Hospital of Philadelphia institutional review board deemed it exempt from the need for approval and informed consent. The study followed the Strengthening the Reporting of Observational Studies in Epidemiology (STROBE) reporting guideline.

### Study Design and Eligibility and Identification of Case Children

The study included 4 retrospective cohorts. Each was assembled on the basis of 1 of 4 conditions: substantial prematurity (prematurity cohort, defined as infants born at ≤30 weeks’ gestational age or with a birth weight <1500 g), critical congenital heart disease (cardiac cohort, defined as newborns with critical congenital heart defects who typically underwent surgery by 1 year of age), oncologic disease (oncologic cohort, defined as children aged 0-18 years with new-onset pediatric oncologic diagnoses, including liquid, solid, and brain cancer), and severe and progressive neurologic impairment (neurologic cohort, defined as children aged 0-18 years with conditions that resulted in severe neurologic impairments associated with substantial functional impairment and with prognosis of progressive deterioration with a substantially shortened life span).

Case children were identified based on the occurrence of an *International Classification of Diseases, Ninth Revision, Clinical Modification* or *International Statistical Classification of Diseases, Tenth Revision, Clinical Modification* (*ICD-10-CM*) diagnosis code in their claim data between July 1, 2015, and June 30, 2016. A full list of the specific codes is provided in eTable 1 in the Supplement. The cohort observational data were health insurance claims observed from July 1, 2015, to December 31, 2017.

### Matching of Case Children to Control Children

Each case child was matched with up to 4 control children based on the case child’s date of birth (matched children had a birth date within 1 month of the case child’s). For each cohort, the set of potential control children included all children who did not have any of the diagnoses for that specific cohort (but could have other diagnoses); individual control children were selected only once.

### Specification of Family Members of Case and Control Children

Family members were operationally defined as individuals with insurance coverage through the policy holder. The policy holder was the parent of either the case or the control child and lived at the same residential address. Family members of case or control children were identified as parents (any age) and siblings (0 to 19 years of age) of the child. For both case and control family members, their initial study day was inherited from the originating case child’s initial study date.

### Specification of Diagnoses, Prescriptions, and Health Care Encounters

Claims files included information regarding diagnoses, prescriptions, and health care encounters. All diagnoses were recorded as *ICD-10-CM* codes. Codes from F10 to F59 and from F50 to F98 (for siblings only) were classified as mental health diagnoses, codes from S00 to T79 and from V00 to Y38 were classified as physical trauma diagnoses, and all other codes were classified as physical health diagnoses.

Prescription information included generic drug names, which were matched to the Anatomical Therapeutic Category coding system via the application program interface using RxMix, version 2.1.16.^34^ Drugs with Anatomical Therapeutic Category codes with prefixes of N05A, N05B (excluding N05BB), N05C, N06A, N06C (excluding N05CM), and N03AE were specified as mental health prescriptions, whereas the remainder were specified as all other prescriptions. Health care encounters in the claims data included categories for hospitalizations, emergency department visits, and urgent care visits.

The composite outcomes of interest were the sums of the number of occurrences of each outcome event type for each individual during the observation period. Similar to the health care utilization data, demographic data, including age, sex, and race and ethnicity, were derived from the insurance database.

### Statistical Analysis

To characterize cohort members, we used descriptive statistics with a 2-tailed *t* test and χ^2^ test to screen for demographic differences between case and control individuals. We specified 4 main overall hypotheses with regard to whether mothers, fathers, sisters, and brothers of case patients, compared with control patients’ family members, experienced increased rates of a composite measure of health care use, diagnoses, and prescriptions, implementing separate models for each of the 4 types of family members. In planned subanalyses, we also analyzed each of the 3 outcome types (health care use, diagnoses, and prescriptions) separately, with further subanalyses within each of these outcome types. In addition, we examined differences between bereaved case parents (identified by noting the death of their child as recorded in the child's claims file) and control parents based on diagnosis. A multivariable negative binomial regression model with a logarithm link function was used to estimate the incidence rate ratios (IRRs) with 95% CIs for case individuals compared with control individuals on the person-level count data for each of the 4 cohorts. All implementations of this model adjusted for individuals’ duration of time observed, age, and the race and ethnicity category specified in the data source (which included a combined category for other race or ethnicity or data missing for race and ethnicity) and accounted for any within-family clustering of sibling observations. Two-sided *P* = .01 was designated as the threshold of statistical significance of the 4 overall hypotheses, and 2-sided *P* = .05 was designated as the threshold of statistical significance for the subanalysis comparisons. Data were analyzed between August 2020 and March 2021. All statistical analyses were performed with Stata, version 16.1 (StataCorp LLC).

A more complete description of the methods is shown in the eAppendix in the Supplement. Discrepancies between the original study protocol^35^ and how the study was conducted are reported in eTable 2 in the Supplement.

## Results

Of the 25 528 total children in the 4 cohorts of pediatric LTCs (eTable 3 in the Supplement), 6909 (27.1%) were case children and 18 619 (72.9%) were control children. Ages ranged from birth to 19 years, with a median age of 6.0 years (IQR, 1-13 years); 13 294 children (52.1%) were male, 1357 (5.3%) were Asian, 1598 (6.3%) were Black, 2448 (9.6%) were Hispanic, 16 893 (66.2%) were White, and 3323 (13.0%) identified as other race or ethnicity or had missing data on race and ethnicity.

Among the 43 357 total parents (Table 1), of whom 11 586 (26.7%) were case parents and 31 771 (73.3%) were control parents, the mean (SD) age was 40.4 (8.1) years; 22 318 (51.5%) were female, 2633 (6.1%) were Asian, 2729 (6.3%) were Black, 4397 (10.1%) were Hispanic, 31 285 (72.2%) were White, and 2313 (5.3%) identified as other race or ethnicity or had missing data on race and ethnicity.

**Table 1.** Demographic Characteristics of Case and Control Parents by Cohort

Characteristics	Parents, No. (%)		*P* value
	Case cohort	Control cohort	
**Prematurity cohort**			
Parents, No.	2093	6062	NA
Age, y			
<19	2 (0.1)	0	.001
19 to <29	204 (9.8)	667 (11.0)	
29 to <39	1407 (67.3)	4178 (68.9)	
39 to <49	435 (20.8)	1140 (18.8)	
≥49	44 (2.1)	77 (1.3)	
Sex			
Female	1158 (55.3)	3151 (52.0)	.009
Male	935 (44.7)	2911 (48.0)	
Race and ethnicity			
Asian	115 (5.5)	391 (6.5)	<.001
Black	243 (11.6)	397 (6.6)	
Hispanic	248 (11.9)	564 (9.3)	
White	1345 (64.3)	4269 (70.4)	
Missing or other	142 (6.8)	441 (7.3)	
**Cardiac cohort**			
Parents, No.	1610	4773	
Age, y			
<19	2 (0.1)	0	.01
19 to <29	165 (10.3)	535 (11.2)	
29 to <39	1072 (66.6)	3266 (68.6)	
39 to <49	339 (21.1)	907 (19.0)	
≥49	31 (1.9)	65 (1.4)	
Sex			
Female	860 (53.5)	2943 (52.3)	.41
Male	749 (46.6)	2279 (47.8)	
Race and ethnicity			
Asian	98 (6.1)	307 (6.4)	.005
Black	153 (9.5)	325 (6.8)	
Hispanic	152 (9.5)	421 (8.8)	
White	1109 (68.9)	3386 (70.9)	
Missing or other	98 (6.1)	334 (7.0)	
**Oncologic cohort**			
Parents, No.	2655	6502	NA
Age, y			
<19	10 (0.4)	0	<.001
19 to <29	30 (1.1)	114 (1.8)	
29 to <39	698 (26.3)	1513 (23.3)	
39 to <49	1297 (48.9)	3154 (48.5)	
≥49	620 (23.4)	1721 (26.5)	
Sex			
Female	1344 (50.6)	3295 (50.7)	.96
Male	1311 (49.4)	3207 (49.3)	
Race and ethnicity			
Asian	159 (6.0)	381 (5.9)	.12
Black	114 (4.3)	363 (5.6)	
Hispanic	279 (10.4)	667 (10.3)	
White	1969 (74.1)	4781 (73.5)	
Missing or other	138 (5.2)	310 (4.8)	
**Neurologic cohort**			
Parents, No.	5679	14 434	NA
Age, y			
<19	12 (0.2)	1 (0.01)	<.001
19 to <29	114 (2.0)	390 (2.7)	
29 to <39	1574 (27.7)	3910 (27.1)	
39 to <49	2651 (46.7)	6590 (45.7)	
≥49	1328 (23.4)	3543 (24.6)	
Sex			
Female	2879 (50.7)	7375 (51.1)	.61
Male	2800 (49.3)	7059 (48.9)	
Race and ethnicity			
Asian	293 (5.2)	919 (6.4)	<.001
Black	316 (5.6)	863 (6.0)	
Hispanic	659 (11.6)	1466 (10.2)	
White	4139 (72.9)	10 580 (73.3)	
Missing or other	273 (4.8)	606 (4.2)	

Abbreviation: NA, not applicable.

Among the 25 706 total siblings (Table 2), of whom 7664 (29.8%) were case siblings and 18 042 (70.2%) were control siblings, the mean (SD) age was 12.1 (6.5) years; 13 114 (51.0%) were male, 1069 (4.2%) were Asian, 1626 (6.3%) were Black, 2690 (10.5%) were Hispanic, 17 681 (68.8%) were White, and 2640 (10.3%) identified as other race or ethnicity or had missing data on race and ethnicity.

**Table 2.** Demographic Characteristics of Case and Control Siblings by Cohort

Characteristics	Siblings, No. (%)		*P* value
	Case cohort	Control cohort	
**Prematurity cohort**			
Siblings, No.	784	2807	NA
Age, y			
0 to <3	30 (3.8)	73 (2.6)	<.001
3 to <5	164 (20.9)	887 (31.6)	
≥5	590 (75.3)	1847 (65.8)	
Sex			
Female	373 (47.6)	1383 (49.3)	.40
Male	411 (52.4)	1424 (50.7)	
Race and ethnicity			
Asian	24 (3.1)	131 (4.7)	<.001
Black	88 (11.2)	167 (6.0)	
Hispanic	93 (11.9)	281 (10.0)	
White	456 (58.2)	1845 (65.7)	
Missing or other	123 (15.7)	383 (13.6)	
**Cardiac cohort**			
Siblings, No.	770	2292	NA
Age, y			
<19	25 (3.3)	51 (2.2)	<.001
19 to <29	207 (26.9)	776 (33.9)	
≥29	538 (69.9)	1465 (63.9)	
Sex			
Female	413 (53.6)	1135 (49.5)	.048
Male	357 (46.4)	1157 (50.5)	
Race and ethnicity			
Asian	30 (3.9)	95 (4.1)	.18
Black	72 (9.4)	153 (6.7)	
Hispanic	76 (9.9)	226 (9.9)	
White	496 (64.4)	1536 (67.0)	
Missing or other	96 (12.5)	282 (12.3)	
**Oncologic cohort**			
Siblings, No.	2072	4058	NA
Age, y			
0 to <3	75 (3.6)	35 (0.9)	<.001
3 to <5	98 (4.7)	147 (3.6)	
≥5	1899 (91.7)	3876 (95.5)	
Sex			
Female	1020 (49.2)	1915 (47.2)	.13
Male	1052 (50.8)	2143 (52.8)	
Race and ethnicity			
Asian	64 (3.1)	163 (4.0)	.30
Black	103 (5.0)	219 (5.4)	
Hispanic	231 (11.2)	413 (10.2)	
White	1467 (70.8)	2860 (9.9)	
Missing or other	207 (10.0)	403 (9.9)	
**Neurologic cohort**			
Siblings, No.	4292	8885	NA
Age, y			
0 to <3	171 (4.0)	117 (1.3)	<.001
3 to <5	293 (6.8)	408 (4.6)	
≥5	3828 (89.2)	8360 (94.1)	
Sex			
Female	2145 (50.0)	4330 (48.7)	.18
Male	2147 (50.0)	4555 (51.3)	
Race and ethnicity			
Asian	136 (3.2)	436 (4.9)	<.001
Black	265 (6.2)	590 (6.6)	
Hispanic	477 (11.1)	922 (10.4)	
White	3024 (70.5)	6150 (69.2)	
Missing or other	390 (9.1)	787 (8.9)	

Abbreviation: NA, not applicable.

In all cohorts, the mean (SD) follow-up time was 612.9 (228.2) days (range, 31-914 days). In total, 491 case children died during the study: 139 of 1176 (11.8%) in the prematurity cohort, 135 of 1520 (8.9%) in the oncologic cohort, 71 of 911 (7.8%) in the cardiac cohort, and 146 of 3302 (4.4%) in the neurologic cohort. Among the parents and the siblings (Table 1 and Table 2), statistically significant differences between case and control families were noted across the 4 cohorts with regard to sex, age, and race and ethnicity categories.

### Parents

Overall, case mothers, compared with control mothers, had 61% higher rates of the combined 3 outcome measures (health care use, diagnoses, and prescriptions) (IRR, 1.61; 95% CI, 1.54-1.68; *P* < .001) after stratifying each of the outcome measures and adjusting for the mothers' ages, duration of observation, race, and ethnicity. Overall, in the same analysis, case fathers, compared with control family fathers, had 55% higher rates of the combined 3 outcome measures (IRR, 1.55; 95% CI, 1.46-1.64; *P* < .001).

**Figure 1.**
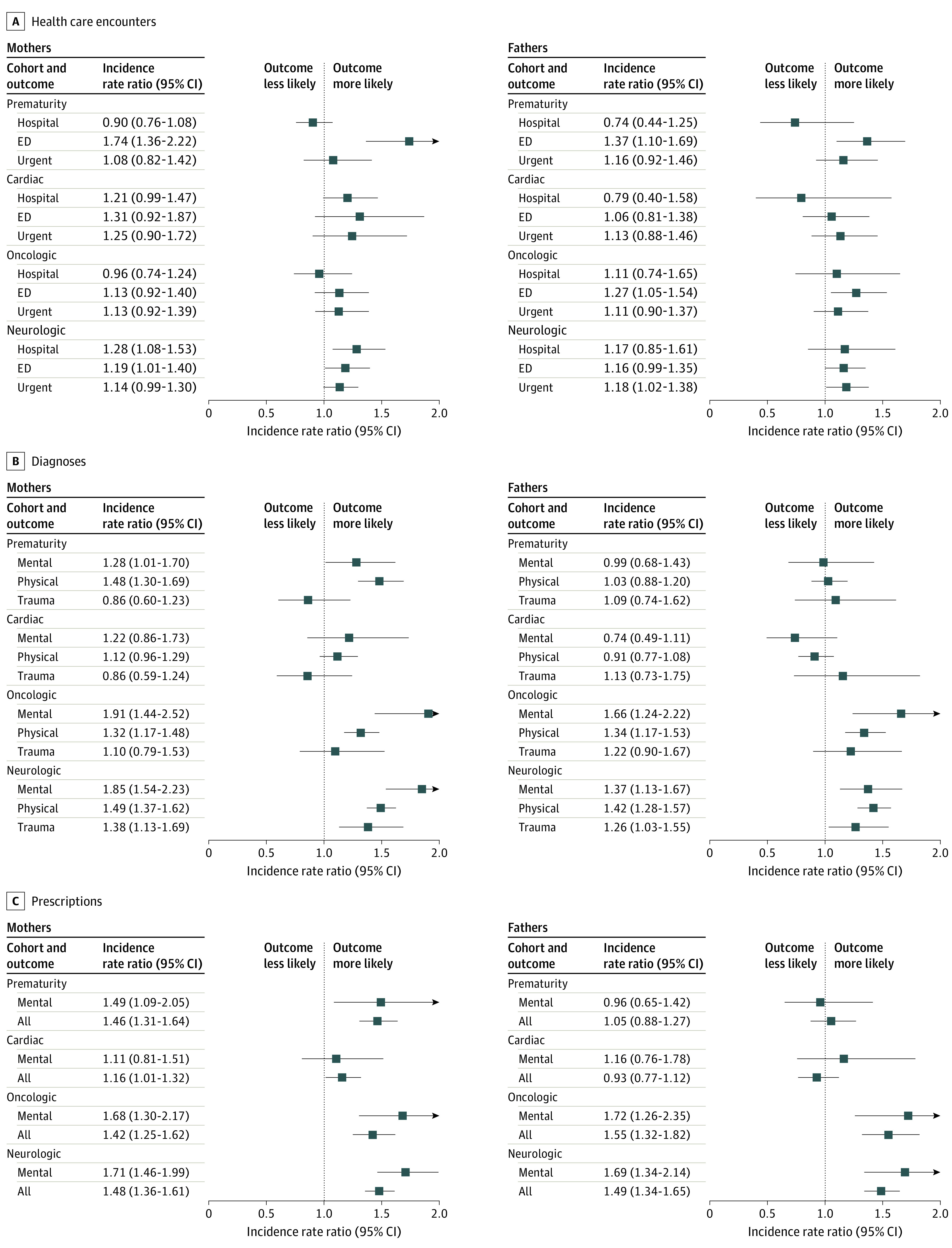
Diagnoses, Prescriptions, and Health Care Encounter Types for Case Parents Compared With Control Parents The analysis was adjusted for parent age, race and ethnicity, and duration of medical coverage. ED indicates emergency department.

To better understand the origins of the overall outcomes for parents in case families compared with control families, we examined each of the 3 major outcome types separately, with further distinctions between subtypes of health care encounters (hospitalizations, emergency department visits, and urgent care visits), diagnoses (mental health, physical health, and physical trauma), and prescriptions (mental health and all other) (Figure 1 and eTable 4 in the Supplement).

With regard to the 3 subtypes of health care encounters, most point estimates (20 of 24 [83.3%]) of the rate ratio of use for case parents compared with control parents indicated that case parents had greater use, and 6 point estimates (25.0%) showed significantly increased use. An exception to this pattern was hospitalizations, for which the point estimates were evenly split between an increase and reduction in use.

With regard to the subtypes of diagnoses, when comparing rate ratio point estimates for case parents with those for control parents, most (19 of 24 [79.2%]) were greater for case parents, and 12 (50.0%) were significantly greater. Increased levels of diagnoses were more consistently seen for the oncologic and neurologic cohorts (12 of 12 [100%]) compared with the prematurity and cardiac cohorts (7 of 12 [58.3%]).

With regard to the subtypes of prescriptions, most of the rate ratio estimates (14 of 16 [87.5%]) were greater for case parents than for control parents, and 11 estimates (68.8%) were significantly greater. Again, the oncologic and neurologic cohorts had a more consistent pattern of increased rate ratio estimates among case parents (8 of 8 [100%], all statistically significant) compared with the prematurity and cardiac cohorts (6 of 8 [75.0%], of which 3 [37.5%] were significantly different).

### Siblings

Overall, sisters of children with LTCs, compared with sisters of children without LTCs, had 68% higher rates of the combined 3 outcome measures (IRR, 1.68; 95% CI, 1.55-1.82; *P* < .001) after adjusting for the sisters' ages, duration of observation, race, and ethnicity and stratifying each of the outcome measures. In the same analysis, brothers of children with LTCs had 70% higher rates than brothers of children without LTCs (IRR, 1.70; 95% CI, 1.56-1.85; *P* < .001).

**Figure 2.**
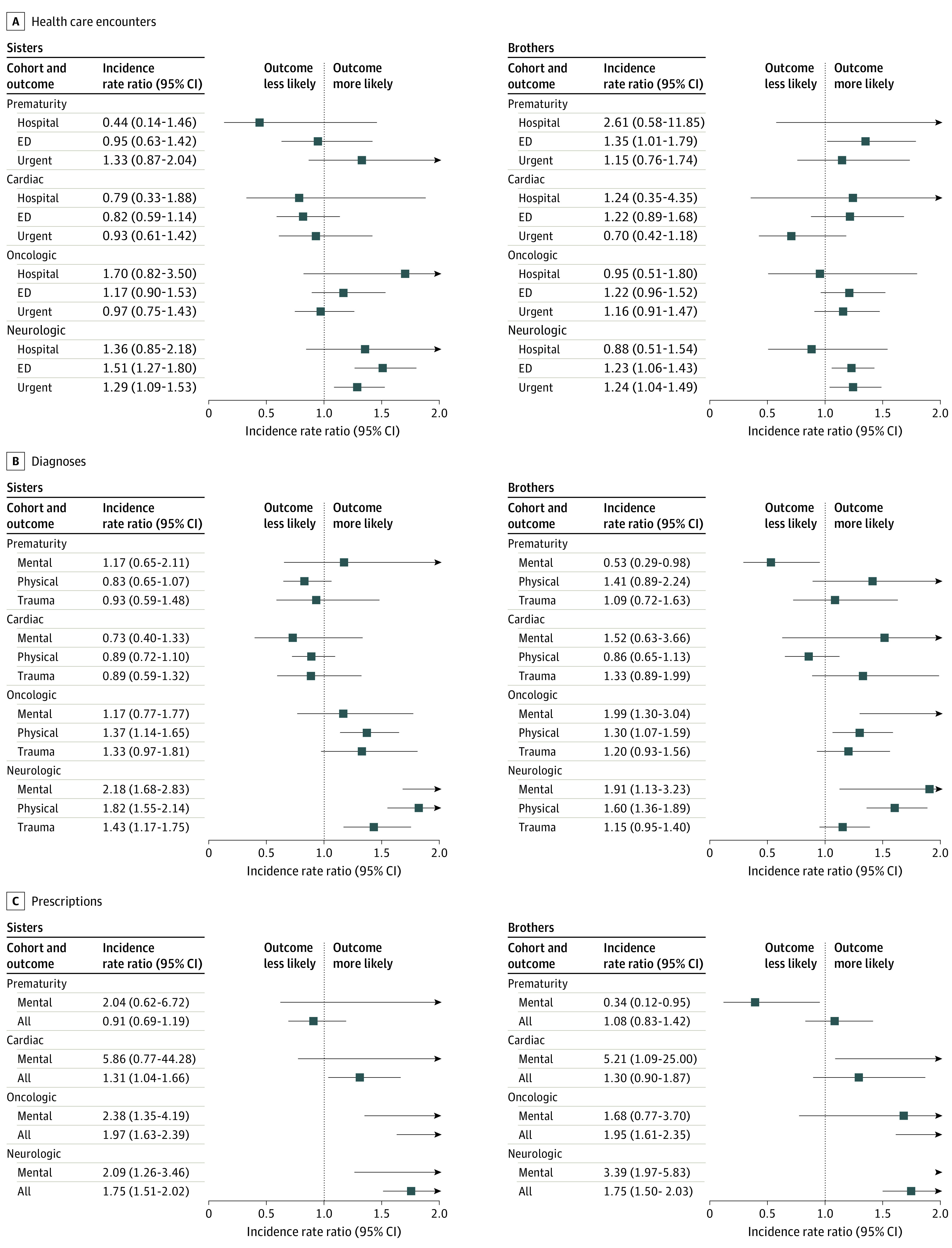
Diagnoses, Prescriptions, and Health Care Encounter Types for Case Siblings Compared With Control Siblings The analysis was adjusted for sibling age, race and ethnicity, and duration of medical coverage. ED indicates emergency department.

Patterns similar to those observed among parents were observed among siblings (Figure 2 and eTable 5 in the Supplement). With regard to health care encounters, most of the rate ratio point estimates (16 of 24 [66.7%]) were greater among case siblings than among control siblings, with 5 (20.8%) being significantly different. With regard to diagnoses, 17 of the 24 point estimates (70.8%) were greater among case siblings, with 8 (33.3%) being significantly different. With regard to prescriptions, 14 of the 16 (87.5%) point estimates were greater among case siblings, with 9 (56.3%) being significantly different. The pattern of greater point estimates among case siblings was more consistently observed in the oncologic and neurologic cohorts than in the prematurity and cardiac cohorts.

### Additional Cross-Cohort, Sex, and Race and Ethnicity Comparisons

We examined the cross-cohort comparative observations in more quantitative detail using a multivariable regression model that stratified the 3 outcomes; controlled for family members’ age, sex, race and ethnicity, and duration of observation; and accounted for family-level clustering of observations (Table 3). The cardiac cohort had the smallest increase among case family members in the composite outcome rate at 29% (IRR, 1.29; 95% CI, 1.15-1.36), followed by the prematurity cohort at 39% (IRR, 1.39; 95% CI, 1.29-1.51), whereas the neurologic cohort had the greatest increase at 76% (IRR, 1.76; 95% CI, 1.68-1.84), followed by the oncologic cohort at 62% (IRR, 1.62; 95% CI, 1.53-1.73).

**Table 3.** Comparison of Composite Outcomes Among Cohorts Overall and by Family Member Type^a^

Type of family member	Cardiac cohort		Prematurity cohort		Oncologic cohort		Neurologic cohort	
	IRR (95% CI)	*P* value	IRR (95% CI)	*P* value	IRR (95% CI)	*P* value	IRR (95% CI)	*P* value
All	1.29 (1.15-1.36)	<.001	1.39 (1.29-1.51)	<.001	1.62 (1.53-1.73)	<.001	1.76 (1.68-1.84)	<.001
Mothers	1.40 (1.24-1.59)	<.001	1.62 (1.47-1.80)	<.001	1.60 (1.45-1.76)	<.001	1.68 (1.57-1.80)	<.001
Fathers	1.13 (0.98-1.29)	.09	1.30 (1.14-1.47)	<.001	1.58 (1.42-1.76)	<.001	1.63 (1.51-1.76)	<.001
Sisters	1.22 (1.02-1.45)	.03	1.05 (0.85-1.31)	.64	1.72 (1.49-1.99)	<.001	2.05 (1.84-2.29)	<.001
Brothers	1.30 (0.96-1.77)	.09	1.27 (0.95-1.70)	.11	1.77 (1.52-2.07)	<.001	1.89 (1.68-2.13)	<.001

Abbreviation: IRR, incident rate ratio.

^a^ Negative binomial regression models were stratified by outcome type (encounters, diagnoses, and prescriptions) and adjusted for the family member’s age, race and ethnicity category, and duration of observation; they also accounted for family-level clustering of observations. In addition, the overall model was adjusted for the type of family member.

Although the case family mothers, fathers, sisters, and brothers had similar degrees of increase in outcome rates compared with their control family counterparts, in all 4 cohorts, case mothers had composite outcome rates that were 44% greater than those for case fathers (IRR, 1.44; 95% CI, 1.39-1.49), whereas case sisters’ composite outcome rates were 11% higher than those for case brothers (IRR, 1.11; 95% CI, 1.05-1.17).

The models adjusted for differences observed in the distribution of the 5 different categories of race and ethnicity among case and control families. In the overall outcomes model including all 4 cohorts, compared with family members classified as White, family members classified as Black had similar composite outcome rates (IRR, 0.98; 95% CI, 0.92-1.05); those classified as Hispanic had lower rates (IRR, 0.90; 95% CI, 0.85-0.95), as did those classified as other race and ethnicity or who had missing data on race and ethnicity (IRR, 0.89; 95% CI, 0.84-0.95). Those classified as Asian had the lowest rates (IRR, 0.66; 95% CI, 0.62-0.71).

### Additional Analysis Restricted to Case Families of Children Who Died

We performed the aforementioned analyses but limited them to the 434 case mothers, 350 case fathers, 263 case sisters, and 223 case brothers of children who died, comparing their health care use during the entire observation period (including before and after the death of the child) with that of the matched control family members (eTable 6 in the Supplement). Overall, the composite rate of health care utilization among case families of children who died was increased 83% (IRR, 1.83; 95% CI, 1.66-2.03) compared with that of control families.

## Discussion

In this large sample of families with children who were born substantially prematurely, were born with critical congenital heart conditions, developed cancer, or had progressive neurologic conditions, we found that parents and siblings of children with these serious illnesses were 55% to 70% more likely to use health care and to receive diagnoses and prescriptions than were family members of control children. This degree of increase among case families compared with control families was similar for mothers and fathers and for sisters and brothers; however, case mothers experienced 44% higher composite outcome rates than did case fathers. Among the 4 different sets of medical conditions, the increases were greatest for neurologic and oncologic case families (range, 62%-76%) and were lower for cardiac and prematurity case families (range, 29%-39%). The degree of increase was greater in families of children who died.

Our findings are consistent with those of previous research. Literature based mostly on self-reported health ratings and health-related quality of life measures^4,36,37^ has documented increases in emotional distress, physical health problems, and sleep disturbances and a lower quality of life among parents of children with LTCs.^2,9,10,11,12,13,14^ This study’s findings are also consistent with those of prior studies showing that siblings of children with LTCs may experience increased stress, depression, anxiety, and behavioral problems and a lower quality of life.^19,21,22^

More recently, studies^38,39^ have examined health care encounters, diagnoses, and prescriptions. One study^38^ found that parents of children who had been recently discharged from a pediatric intensive care unit were more than twice as likely to receive a mental health diagnosis during the subsequent 6 months compared with during the prior 6 months, and 3% to 4% of these parents received new prescriptions for antidepressant or anxiolytic medications, with mothers twice as likely as fathers to receive new prescriptions. A study^39^ of mothers of children with debilitating conditions residing in England identified an increased risk among these mothers (compared with control mothers) for depression, cardiovascular disease, and death.

Three aspects of the current study’s findings warrant discussion. First, health care encounters, diagnoses, and prescriptions are predicated on health care use and practices, and thus the association between the study’s results and the actual physical and mental health of these family members is indirect. If the case families of children with LTCs were less likely to use health care than were control families (as has been observed for families of children who have cancer^19^), then this study’s findings likely underestimate the degree of increase of physical and mental health conditions in the case families. In contrast, parents of children with LTCs may be more likely to use health care, for instance, because parents often worry that the siblings will have health problems because of the ill child’s LTC.^40^ This pattern of behavior would result in our having overestimated the true degree of increased health care use. Of these 2 possibilities, the findings that case families also showed greater increases in diagnoses and prescriptions than did control families and that the increases in diagnoses and prescriptions were greater than for health care encounters suggest that case families may have had more health problems but may have been less likely to seek health care when needed compared with control families.

Second, comparisons of the study’s findings among the 4 cohorts may provide insights regarding specific theories about how having a child with an LTC in the family could adversely affect the physical and mental health of parents and siblings. Despite findings in the cohort of families of children who died that suggested an association between a child’s death and increased rates of the study outcomes, the greater degree of increases in outcome rates in the oncologic and neurologic cohorts cannot be explained by this factor because the mortality was significantly higher in the prematurity cohort. An alternative explanation focuses on the onset, duration, and prognostic uncertainty of serious illness as well as the parental workload.^41^ Compared with case newborn infants in the cardiac and prematurity cohorts, most of whose conditions likely substantially improved after several months (thereby resolving prognostic uncertainty), children in the neurologic and oncologic cohorts were most likely previously healthy and more likely to experience long-duration illnesses with sustained prognostic uncertainty and substantial ongoing parental workload, such that these family members likely experienced the stress and other negative effects associated with a child’s LTC for longer periods. These observations would be consistent with at least 3 theories of how having an ill family member may affect other family members. One theory is that the emotional, financial, and other forms of stress of having an ill family member impose a deleterious allosteric load on other family members in the short and long terms.^11,42,43,44,45^ The second theory is that over time, maladaptation to this load would result in poor health habits.^6,12^ The third theory is that coping with the extra tasks and constraints (including financial hardship^46^) imposed by the care needs of the ill family member may lead other family members to defer preventive care.^16,47,48,49,50^

Third, if this study’s findings, in conjunction with previous findings, are accepted as indicating higher levels of physical and mental health conditions among parents and siblings of children with LTCs, questions arise regarding whether this adverse health effect can be prevented or mitigated by effective physical and mental health care. Care for these at-risk families should also provide both instrumental support (eg, assistance with transportation, insurance, navigation, financial hardship, health promotion, and school) and emotional support (eg, psychotherapy, support groups, and stress reduction) aimed to minimize financial hardship and distress.

### Limitations

This study has limitations. First, the study used data generated from health care encounters and did not directly measure differences in physical and mental health. Second, the sample consisted entirely of families who had private insurance coverage, limiting generalizability to individuals without private insurance coverage. Third, because only children with 1 of 4 LTCs were included, the details of these findings cannot be generalized to all families who have children with any form of LTC.

## Conclusions

The findings of this cohort study, limited to families of children with 1 of 4 LTCs, are consistent with increasing evidence^2,4^ that family members of children with LTCs may have increased health care use and poorer mental and physical health. Although more research is warranted to better understand the mechanisms underlying these findings, interventions for parents and siblings of children with LTCs that aim to safeguard their mental and physical well-being appear to be warranted.

## Notes

Supplement. **eAppendix.** Expanded Description of Study Methods **eTable 1.** International Classification of Disease Codes Used to Define Cohorts and Identify Case Patients With Specified Conditions **eTable 2.** Discrepancies From Proposed Analysis Registered on ClinicalTrials.gov **eTable 3.** Demographic Characteristics of Case and Matched Control Children by Cohort **eTable 4.** Comparison of Mothers and Fathers Regarding Health Care Encounters, Diagnoses, and Prescriptions **eTable 5.** Comparison of Sisters and Brothers Regarding Health Care Encounters, Diagnoses, and Prescriptions **eTable 6.** Comparison of Bereaved Mothers and Fathers Regarding Health Care Encounters, Diagnoses, and Prescriptions

## References

[1] Zimmermann K, Bergstraesser E, Engberg S, ; PELICAN Consortium. When parents face the death of their child: a nationwide cross-sectional survey of parental perspectives on their child’s end-of life care. BMC Palliat Care. 2016;15(30):30. doi:10.1186/s12904-016-0098-3 26956995 PMC4784404

[2] Bally JMG, Smith NR, Holtslander L, . A metasynthesis: uncovering what is known about the experiences of families with children who have life-limiting and life-threatening illnesses. J Pediatr Nurs. 2018;38:88-98. doi:10.1016/j.pedn.2017.11.004 29357986

[3] Humphrey LM, Hill DL, Carroll KW, Rourke M, Kang TI, Feudtner C. Psychological well-being and family environment of siblings of children with life threatening illness. J Palliat Med. 2015;18(11):981-984. doi:10.1089/jpm.2015.0150 26393493

[4] Parker R, Houghton S, Bichard E, McKeever S. Impact of congenital heart disease on siblings: a review. J Child Health Care. 2020;24(2):297-316. doi:10.1177/1367493520914738 32216565 PMC7488830

[5] Silva-Rodrigues FM, Pan R, Pacciulio Sposito AM, de Andrade Alvarenga W, Nascimento LC. Childhood cancer: impact on parents' marital dynamics. Eur J Oncol Nurs. 2016;23:34-42. 10.1016/j.ejon.2016.03.002 27456373

[6] Wiener L, Viola A, Kearney J, ; Lone Parent Study Group. Impact of caregiving for a child with cancer on parental health behaviors, relationship quality, and spiritual faith: do lone parents fare worse? J Pediatr Oncol Nurs. 2016;33(5):378-386. doi:10.1177/1043454215616610 26668211 PMC5066587

[7] Brooten D, Youngblut JM, Caicedo C, Del Moral T, Cantwell GP, Totapally B. Parents’ acute illnesses, hospitalizations, and medication changes during the difficult first year after infant or child NICU/PICU death. Am J Hosp Palliat Care. 2018;35(1):75-82. doi:10.1177/1049909116678597 27852818 PMC5344737

[8] Rosenberg AR, Wolfe J, Bradford MC, . Resilience and psychosocial outcomes in parents of children with cancer. Pediatr Blood Cancer. 2014;61(3):552-557. doi:10.1002/pbc.24854 24249426 PMC4066960

[9] Meltzer LJ, Mindell JA. Relationship between child sleep disturbances and maternal sleep, mood, and parenting stress: a pilot study. J Fam Psychol. 2007;21(1):67-73. doi:10.1037/0893-3200.21.1.67 17371111

[10] Kuster PA, Badr LK. Mental health of mothers caring for ventilator-assisted children at home. Issues Ment Health Nurs. 2006;27(8):817-835. doi:10.1080/01612840600840588 16938786

[11] Oxley R. Parents’ experiences of their child’s admission to paediatric intensive care. Nurs Child Young People. 2015;27(4):16-21. doi:10.7748/ncyp.27.4.16.e564 25959486

[12] Placencia FX, McCullough LB. Biopsychosocial risks of parental care for high-risk neonates: implications for evidence-based parental counseling. J Perinatol. 2012;32(5):381-386. doi:10.1038/jp.2011.109 21904297

[13] Vrijmoet-Wiersma CM, van Klink JMM, Kolk AM, Koopman HM, Ball LM, Maarten Egeler R. Assessment of parental psychological stress in pediatric cancer: a review. J Pediatr Psychol. 2008;33(7):694-706. doi:10.1093/jpepsy/jsn007 18287109

[14] Woolf C, Muscara F, Anderson VA, McCarthy MC. Early traumatic stress responses in parents following a serious illness in their child: a systematic review. J Clin Psychol Med Settings. 2016;23(1):53-66. doi:10.1007/s10880-015-9430-y 26296614

[15] Boyden JY, Hill DL, Nye RT, ; PPCRN SHARE Project Group. Pediatric palliative care parents’ distress, financial difficulty, and child symptoms. J Pain Symptom Manage. 2021;S0885-3924(21)00492-9. doi:10.1016/j.jpainsymman.2021.08.004 34425212 PMC8816828

[16] Ratliffe CE, Harrigan RC, Haley J, Tse A, Olson T. Stress in families with medically fragile children. Issues Compr Pediatr Nurs. 2002;25(3):167-188. doi:10.1080/01460860290042558 12230829

[17] Kearney JA, Salley CG, Muriel AC. Standards of psychosocial care for parents of children with cancer. Pediatr Blood Cancer. 2015;62(suppl 5):S632-S683. doi:10.1002/pbc.25761 26700921 PMC5066591

[18] Diaz-Caneja A, Gledhill J, Weaver T, Nadel S, Garralda E. A child’s admission to hospital: a qualitative study examining the experiences of parents. Intensive Care Med. 2005;31(9):1248-1254. doi:10.1007/s00134-005-2728-8 16021417

[19] Zeltzer LK, Dolgin MJ, Sahler OJ, . Sibling adaptation to childhood cancer collaborative study: health outcomes of siblings of children with cancer. Med Pediatr Oncol. 1996;27(2):98-107. doi:10.1002/(SICI)1096-911X(199608)27:2<98::AID-MPO6>3.0.CO;2-O 8649327

[20] Houtzager BA, Grootenhuis MA, Last BF. Supportive groups for siblings of pediatric oncology patients: impact on anxiety. Psychooncology. 2001;10(4):315-324. doi:10.1002/pon.528 11462230

[21] Houtzager BA, Grootenhuis MA, Caron HN, Last BF. Quality of life and psychological adaptation in siblings of paediatric cancer patients, 2 years after diagnosis. Psychooncology. 2004;13(8):499-511. doi:10.1002/pon.759 15295772

[22] Houtzager BA, Grootenhuis MA, Last BF. Adjustment of siblings to childhood cancer: a literature review. Support Care Cancer. 1999;7(5):302-320. doi:10.1007/s005200050268 10483815

[23] Holditch-Davis D, Bartlett TR, Blickman AL, Miles MS. Posttraumatic stress symptoms in mothers of premature infants. J Obstet Gynecol Neonatal Nurs. 2003;32(2):161-171. doi:10.1177/0884217503252035 12685667

[24] Helle N, Barkmann C, Ehrhardt S, von der Wense A, Nestoriuc Y, Bindt C. Postpartum anxiety and adjustment disorders in parents of infants with very low birth weight: cross-sectional results from a controlled multicentre cohort study. J Affect Disord. 2016;194:128-134. doi:10.1016/j.jad.2016.01.016 26820762

[25] Singer LT, Salvator A, Guo S, Collin M, Lilien L, Baley J. Maternal psychological distress and parenting stress after the birth of a very low-birth-weight infant. JAMA. 1999;281(9):799-805. doi:10.1001/jama.281.9.799 10071000 PMC10189739

[26] López R, Frangini P, Ramírez M, . Well-being and agency in parents of children with congenital heart disease: a survey in Chile. World J Pediatr Congenit Heart Surg. 2016;7(2):139-145. doi:10.1177/2150135115623284 26957395

[27] Grønning Dale MT, Solberg Ø, Holmstrøm H, Landolt MA, Eskedal LT, Vollrath ME. Well-being in mothers of children with congenital heart defects: a 3-year follow-up. Qual Life Res. 2013;22(8):2063-2072. doi:10.1007/s11136-012-0326-0 23196922

[28] Wei H, Roscigno CI, Hanson CC, Swanson KM. Families of children with congenital heart disease: a literature review. Heart Lung. 2015;44(6):494-511. doi:10.1016/j.hrtlng.2015.08.005 26404115

[29] Dockerty JD, Williams SM, McGee R, Skegg DCG. Impact of childhood cancer on the mental health of parents. Med Pediatr Oncol. 2000;35(5):475-483. doi:10.1002/1096-911X(20001101)35:5<475::AID-MPO6>3.0.CO;2-U 11070480

[30] Klassen AF, Klaassen R, Dix D, . Impact of caring for a child with cancer on parents’ health-related quality of life. J Clin Oncol. 2008;26(36):5884-5889. doi:10.1200/JCO.2007.15.2835 19029424

[31] Byrne MB, Hurley DA, Daly L, Cunningham CG. Health status of caregivers of children with cerebral palsy. Child Care Health Dev. 2010;36(5):696-702. doi:10.1111/j.1365-2214.2009.01047.x 20074250

[32] Mori Y, Downs J, Wong K, Leonard H. Longitudinal effects of caregiving on parental well-being: the example of Rett syndrome, a severe neurological disorder. Eur Child Adolesc Psychiatry. 2019;28(4):505-520. doi:10.1007/s00787-018-1214-0 30151799

[33] Qian Y, McGraw S, Henne J, Jarecki J, Hobby K, Yeh W-S. Understanding the experiences and needs of individuals with spinal muscular atrophy and their parents: a qualitative study. BMC Neurol. 2015;15(1):217. doi:10.1186/s12883-015-0473-3 26499462 PMC4619513

[34] National Library of Medicine, National Institutes of Health. RxMix: creating applications from NLM drug APIs. Accessed May 1, 2021. https://mor.nlm.nih.gov/RxMix/

[35] Impact of serious pediatric illness on parent and sibling health. Published 2019. Accessed November 17, 2021. https://clinicaltrials.gov/ct2/show/NCT03971344

[36] Morhun JM, Racine NM, Guilcher GMT, Tomfohr-Madsen LM, Schulte FSM. Health-related quality of life and well-being in parents of infants and toddlers with cancer. Curr Oncol. 2020;27(2):e206-e215. doi:10.3747/co.27.4937 32489270 PMC7253729

[37] Drotar D, Hack M, Taylor G, Schluchter M, Andreias L, Klein N. The impact of extremely low birth weight on the families of school-aged children. Pediatrics. 2006;117(6):2006-2013. doi:10.1542/peds.2005-2118 16740842

[38] Logan GE, Sahrmann JM, Gu H, Hartman ME. Parental mental health care after their child’s pediatric intensive care hospitalization. Pediatr Crit Care Med. 2020;21(11):941-948. doi:10.1097/PCC.0000000000002559 32947380 PMC7609586

[39] Fraser LK, Murtagh FE, Aldridge J, Sheldon T, Gilbody S, Hewitt C. Health of mothers of children with a life-limiting condition: a comparative cohort study. Arch Dis Child. 2021;106(10):987-993. doi:10.1136/archdischild-2020-320655 33653713 PMC8461446

[40] Ballard KL. Meeting the needs of siblings of children with cancer. Pediatr Nurs. 2004;30(5):394-401. 15587532

[41] Hexem KR, Bosk AM, Feudtner C. The dynamic system of parental work of care for children with special health care needs: a conceptual model to guide quality improvement efforts. BMC Pediatr. 2011;11:95. doi:10.1186/1471-2431-11-95 22026518 PMC3234186

[42] Ader R, Cohen N, Felten D. Psychoneuroimmunology: interactions between the nervous system and the immune system. Lancet. 1995;345(8942):99-103. doi:10.1016/S0140-6736(95)90066-7 7815892

[43] Kuster PA, Merkle CJ. Caregiving stress, immune function, and health: implications for research with parents of medically fragile children. Issues Compr Pediatr Nurs. 2004;27(4):257-276. doi:10.1080/01460860490884165 15764433

[44] Rohleder N, Marin TJ, Ma R, Miller GE. Biologic cost of caring for a cancer patient: dysregulation of pro- and anti-inflammatory signaling pathways. J Clin Oncol. 2009;27(18):2909-2915. doi:10.1200/JCO.2008.18.7435 19433690

[45] Vitaliano PP, Zhang J, Scanlan JM. Is caregiving hazardous to one’s physical health? a meta-analysis. Psychol Bull. 2003;129(6):946-972. doi:10.1037/0033-2909.129.6.946 14599289

[46] Foster CC, Chorniy A, Kwon S, Kan K, Heard-Garris N, Davis MM. Children with special health care needs and forgone family employment. Pediatrics. 2021;148(3):e2020035378. doi:10.1542/peds.2020-035378 34433691 PMC9219960

[47] Bilodeau M, Ma C, Al-Sayegh H, Wolfe J, Bona K. Household material hardship in families of children post-chemotherapy. Pediatr Blood Cancer. 2018;65(1). doi:10.1002/pbc.26743 28941160 PMC6042835

[48] Thomson J, Shah SS, Simmons JM, . Financial and social hardships in families of children with medical complexity. J Pediatr. 2016;172:187-193. doi:10.1016/j.jpeds.2016.01.049 26897040 PMC4846519

[49] Bona K, London WB, Guo D, Frank DA, Wolfe J. Trajectory of material hardship and income poverty in families of children undergoing chemotherapy: a prospective cohort study. Pediatr Blood Cancer. 2016;63(1):105-111. doi:10.1002/pbc.25762 26398865

[50] Ilowite MF, Al-Sayegh H, Ma C, . The relationship between household income and patient-reported symptom distress and quality of life in children with advanced cancer: a report from the PediQUEST study. Cancer. 2018;124(19):3934-3941. doi:10.1002/cncr.31668 30216416 PMC6561342

