# Supplementary material for: Association Between Children With Life-Threatening Conditions and Their Parents’ and Siblings’ Mental and Physical Health
Source: JAMA Netw Open. 2021 Dec 20;4(12):e2137250. doi: 10.1001/jamanetworkopen.2021.37250 (PMC8689391; doi:10.1001/jamanetworkopen.2021.37250)
Supplement: Supplement. — eAppendix. Expanded Description of Study Methods eTable 1. International Classification of Disease Codes Used to Define Cohorts and Identify Case Patients With Specified Conditions eTable 2. Discrepancies From Proposed Analysis Registered on ClinicalTrials.gov eTable 3. Demographic Characteristics of Case and Matched Control Children by Cohort eTable 4. Comparison of Mothers and Fathers Regarding Health Care Encounters, Diagnoses, and Prescriptions eTable 5. Comparison of Sisters and Brothers Regarding Health Care Encounters, Diagnoses, and Prescriptions eTable 6. Comparison of Bereaved Mothers and Fathers Regarding Health Care Encounters, Diagnoses, and Prescriptions [file jamanetwopen-e2137250-s001.pdf]

Supplementary Online Content

Feudtner C, Nye RT, Boyden JY, et al. Association between children with life-threatening conditions and their parents' and siblings' mental and physical health. *JAMA Netw Open*. 2021;4(12):e2137250. doi:10.1001/jamanetworkopen.2021.37250

eAppendix. Expanded Description of Study Methods
eTable 1. International Classification of Disease Codes Used to Define Cohorts and Identify Case Patients With Specified Conditions
eTable 2. Discrepancies From Proposed Analysis Registered on ClinicalTrials.gov
eTable 3. Demographic Characteristics of Case and Matched Control Children by Cohort
eTable 4. Comparison of Mothers and Fathers Regarding Health Care Encounters, Diagnoses, and Prescriptions
eTable 5. Comparison of Sisters and Brothers Regarding Health Care Encounters, Diagnoses, and Prescriptions
eTable 6. Comparison of Bereaved Mothers and Fathers Regarding Health Care Encounters, Diagnoses, and Prescriptions

This supplementary material has been provided by the authors to give readers additional information about their work.

© 2021 Feudtner C et al. *JAMA Network Open*.

eAppendix. EXPANDED DESCRIPTION OF STUDY METHODS

Study design and eligibility and identification of case children

The study constructed four retrospective cohorts, each assembled around a case child with one of the four following conditions. Substantial Prematurity (***Prematurity***) cohort: infants born at 30 weeks gestational age or less, or with a birthweight less than 1500 grams. Critical Congenital Heart Disease (***Cardiac***) cohort: newborns with critical congenital heart defects who typically undergo surgery by 12 months of life. Oncology (***Oncologic***) cohort: Children between 0 and 18 years of age with new onset pediatric oncologic diagnoses, including liquid, solid, and brain cancer. Severe and Progressive Neurological Impairment (***Neurologic***) cohort: Children between 0 and 18 years of age with conditions that result in severe neurologic impairments associated with substantial functional impairment, and with prognosis of progressive deterioration with substantially shortened lifespans. For the Prematurity and the Cardiac cohorts, which consisted of newborns, potential case infants who were twins (or higher multiple birth siblings) were excluded.

Case children were identified based on the occurrence of an International Statistical Classification of Diseases and Related Health Problems, 9th or 10th Revision, Clinical Modification (ICD 9 CM and ICD 10 CM, respectively) diagnosis code in their claim data during the interval between 1 July 2015 and 30 June 2016. A full list of the specific codes appears in Supplemental Table A.

© 2021 Feudtner C et al. *JAMA Network Open*.

Newborn infants in the Prematurity and the Cardiac cohorts were, by definition, incidence cases. For these case children, their initial study day of cohort entry was defined as their birth date, falling between 1 July 2015 and 30 June 2016. Infants and children in the Oncology cohort were screened during a 6-month look-back period that extended to 1 January 2015 to exclude patients who had received an oncologic diagnosis prior to 1 July 2015, so that this cohort represents likely incident cases. For these case children, their initial study date of cohort entry was the day on which the first cancer diagnostic code occurred in their claim data. Children in the Neurologic cohort, by contrast, were not screened to exclude diagnosis prior to the study period, so this cohort represents prevalent cases. For these case children, to avoid the definitional designation of a cohort entry day associated with any seasonal variation in the occurrence of health conditions or healthcare utilization, a random day within the 1 July 2015 and 30 June 2016 interval was assigned as their initial study day.

Matching of case children to control children

Each case child was matched with up to four control children based on the case child's date of birth (within a month or less). If more than 4 potential control children were available, 4 control children were selected at random. For each cohort, the set of potential control children included all children who did not have any of the diagnoses for that specific cohort (but could have other diagnoses), who were not multiple birth siblings, and had matching case families with medical coverage; individual control children were selected only once.

Specification of family members of case and the control children

© 2021 Feudtner C et al. *JAMA Network Open*.

Family members were operationally defined as individuals covered via the policy holder. The policy holder was the parent of either the case or the control child and lived at the same residential address. Family members of case children were identified as case parents (any age) and case siblings (0 to 19 years of age), and similarly for family members of the control children. For both case and control family members, their initial study day was inherited from the originating case child's initial study day. Among case families, if no family member had insurance coverage after the initial study day, then the case family and the matching control families were dropped. For control families, if no member had insurance coverage after the initial study day, then that control family was omitted and the case-to-control ratio adjusted.

Cohort observation interval

The cohort observation interval was from 1 July 2015 until 31 December 2017. Families had different lengths of observation time, due to timing of cohort entry and different durations of insurance coverage.

Specification of diagnoses, prescriptions, and healthcare encounters

Claims files during the cohort observation period included information regarding diagnoses, prescriptions, and healthcare encounters. All diagnoses were recorded as ICD-10-CM codes. ICD-10-CM codes in the range from F10 to F59, as well as F50 to F98 for siblings only, were classified as "mental health" diagnoses; codes in the range from S00 to T79 and V00 to Y38 were classified as physical "trauma" diagnoses; all other codes were classified as "physical health" diagnoses.

© 2021 Feudtner C et al. *JAMA Network Open*.

Prescription information included generic drug names. Compound generic drug names were separated into single generic drug component names. The list of all observed generic drug component names was then matched to the Anatomical Therapeutic Category (ATC) coding system via the RxMix API.[31] Drugs matched to ACT codes with prefixes of N05A, N05B (excluding N05BB), N05C, N06A, N06C (excluding N05CM), and N03AE were specified as "mental health" prescriptions, while the remainder were specified as "all other" prescriptions. Healthcare encounters in the claims data included categories for hospitalizations, emergency department (ED) visit, and urgent care visits.

For each cohort member during the observation period, we summed the total count of diagnoses, of prescriptions, and of healthcare encounters, within each of the categories described above.

Statistical Analysis

We specified four main overall hypotheses regarding whether mothers, fathers, sisters, and brothers of case patients, compared to control patients' family members, experience increased rates of a composite measure of healthcare use, diagnoses, and prescriptions, implementing separate models for each of the four types of family members. In planned sub-analyses, we also analyzed each of the three outcome types (healthcare use, diagnoses, and prescriptions) separately, and further sub-analyses within each of these outcome types. Finally, we examined differences in bereaved case parents compared to control parents. Multivariable negative binomial regression model with logarithm link function was used to estimate the incidence rate ratio (IRR) with 95% confidence interval (CI) of case individuals with respect to the control individuals, on the

© 2021 Feudtner C et al. *JAMA Network Open*.

person-level count data for each of the four cohorts separately. All implementations of this model adjusted for individuals' duration of time observed, age, and the race/ethnicity category specified in the data source (which included a category of other or missing) and accounted for any within-family clustering of sibling observations. A P-value of 0.01 was designated as the threshold of statistical significance of the 4 overall hypotheses, and of 0.05 for the sub-analysis comparisons.

Of note, we viewed several possible occurrences as being part of the potential causal pathway between exposure to a child with a LTC and adverse health consequences for other family members. Specifically, if the child were to have died (most likely due to the LTC), the subsequent bereavement is one mechanism by which adverse health outcomes could occur. If a co-parent or one of the other children in the family developed a physical or mental health condition secondary to exposure to the child with the LTC, and this occurrence than had an additional adverse consequence on the co-parent or other siblings, this could also be part of the causal pathway. We therefore did not view these occurrences as confounding per se.

All analyses were performed with Stata version 16.1 (StataCorp LLC, College Station, TX).

This study followed the STROBE cohort checklist when writing our report.[51] The study was registered on ClinicalTrials.Org (NCT03971344)[32,33]; discrepancies between what was registered and how the study was conducted are reported in Supplemental Table B.

© 2021 Feudtner C et al. *JAMA Network Open*.

This study used deidentified data and thus did not constitute human subjects research as defined by The Children's Hospital of Philadelphia Institutional Review Board. Data was provided by the health insurance company, Cigna, based on existing claims data of customers; Cigna served as the safe harbor for the initial data management and deidentification, which included the conversion of all dates to case-child-specific study days (ranging from 0 upward).

© 2021 Feudtner C et al. *JAMA Network Open*.

eTable 1: International Classification of Disease codes used to define cohorts and identify case patients with specified conditions

ICD-9-CM	ICD-10-CM	Description
**Premature birth cohort**		
**765.01**	P07.01	Extreme immaturity, less than 500 grams
**765.02**	P07.02	Extreme immaturity, 500-749 grams
**765.03**	P07.03	Extreme immaturity, 750-999 grams
**765.04**	P07.14	Extreme immaturity, 1000-1249 grams
**765.05**	P07.15	Extreme immaturity, 1250-1499 grams
**765.11**	P07.01	Other preterm infants, less than 500 grams
**765.12**	P07.02	Other preterm infants, 500-749 grams
**765.13**	P07.03	Other preterm infants, 750-999 grams
**765.14**	P07.14	Other preterm infants, 1000-1249 grams
**765.15**	P07.15	Other preterm infants, 1250-1499 grams
**765.21**	P07.21, P07.22	Less than 24 complete weeks
**765.22**	P07.23	24 complete weeks
**765.23**	P07.24, P07.25	25-26 complete weeks
**765.24**	P07.26, P07.31	27-28 complete weeks
**765.25**	P07.32, P07.33	29-30 complete weeks
**Newborns with Critical Congenital Heart Disease**		
**745.1**	Q20.3	Discordant ventriculoarterial connection, complete transposition
**745.2**	Q21.3	Tetrology of Fallot
**746.2**	Q22.5	Ebstein's anomaly
**746.7**	Q23.4	Hypoplastic left heart syndrome
**746.1**	Q22.9	Tricuspid atresia and stenosis, congenital
**747.41**	Q26.2	Total anomalous pulmonary venous return
**747.31**	Q25.5, Q25.71	Pulmonary artery coarctation and atresia
**747.32**	Q25.72	Congenital pulmonary arteriovenous malformation
**747.39**	Q25.6, Q25.79	Other anomalies of pulmonary artery and pulmonary circulation
**747.11**	Q25.21	Interrupted aortic arch
**745.11**	Q20.1	Double-outlet right ventricle

© 2021 Feudtner C et al. *JAMA Network Open*.

**747.1**	Q25.1	Coarctation of the aorta
**745**	Q20.0	Common truncus, common arterial trunk, truncus arteriosus
**New onset oncology cohort**		
**170 – 170.9**	C41.0-C41.4, C41.9, C40.00, C40.10, C40.20, C40.30	Malignant neoplasm of bone and articular cartilage
**191.0 – 191.9**	C71.0-71.9	Malignant neoplasm of brain
**194**	C74.90	Malignant neoplasm of adrenal gland
**201.00 – 201.98**	C81.00-C81.49, C81.70-C81.99	Hodgkin's disease
**204**	C91.00	Acute lymphoid leukemia, without mention of having achieved remission
**205**	C92.00, C92.40, C92.50	Acute myeloid leukemia, without mention of having achieved remission
**Severe neurologic impairment cohort**		
**277.5**	E76.0 to E76.9	Mucopolysaccharidosis
		Disorders of glycosaminoglycan metabolism
**330**		Cerebral degenerations usually manifest in childhood
**330.0**	E70.0 to E75.6	Leukodystrophy
**330.1**		Cerebral lipidoses
**330.2**	G93.89	Cerebral degeneration in generalized lipidoses
**330.3**	G93.9	Cerebral degeneration of childhood in other diseases classified elsewhere
**330.8**	F84.2, G31.81, G31.82	Other specified cerebral degenerations of childhood
**330.9**	G31.9	Unspecified cerebral degenerations of childhood
**334**	G11.1	Friedrich's ataxia
**335**	G12.0	Werdnig-Hoffman disease
**343.2**	G80.0	Congenital quadriplegia
**345.11**	G40.311, G40.411, G40.419	Generalized convulsive epilepsy, with intractable epilepsy
**345.61**	G40.823, G40.824	Infantile spasms, with intractable epilepsy
**345.91**	G40.911, G40.919	Epilepsy, unspecified, with intractable epilepsy

© 2021 Feudtner C et al. *JAMA Network Open*.

eTable 2: Discrepancies from proposed analysis registered on ClinicalTrials.gov

**Proposed**		**Actual**	
Study Attribute	Details	Discrepancy	Comments
**Groups/Cohorts**			
Family members of newborns extremely premature	Parents and siblings (if any) of infants born at 30 weeks gestational age or less, or with a birthweight less than 1500 grams.	No	
Family members of new pediatric oncology patients	Parents and siblings (if any) of patients with new onset (not relapses) pediatric oncologic diagnoses including liquid, solid, and brain cancer.	No	
Family members of critical congenital heart defect patients	Parents and siblings (if any) of newborns with critical congenital heart defects who typically undergo surgery by 12 months of life.	No	
Family members of children with severe neurological impairment	Parents and siblings (if any) of patients with severe neurologic impairments, associated with substantial functional impairment, relentless progressive deterioration, or substantially shortened lifespans.	No	
**Outcomes**			
New mental and physical health diagnoses among siblings and parents	Outcome will be assessed based on diagnoses in de-identified claims data [Time Frame: 3 years]	Partial	In addition, we also assessed for new trauma diagnoses for siblings and parents.
New mental and physical health prescriptions among siblings and parents	Outcome will be assessed based on prescription data in de-identified claims data [Time Frame: 3 years]	No	
Emergency department usage, ambulatory usage, and hospitalizations among parents and siblings	Outcome will be assessed based on encounter data in de-identified claims data [Time Frame: 3 years]	No	
Adherence to chronic disease management standards among parents	Outcome will be assessed based on data in de-identified claims data [Time Frame: 3 years]	Yes	Data was not deemed to be adequate for analysis.
Receipt of well-child visits and immunizations among siblings	Outcome will be assessed based on data in de-identified claims data [Time Frame: 3 years]	Yes	Data was not deemed to be adequate for analysis.
		Yes	To provide quantitative summary findings, we analyzed the composite outcome (diagnoses, prescriptions, and encounters) for both siblings and parents
**Sampling/ Eligibility Criteria**			
Sampling:	Non-probability sample of all sexes, ages, and no volunteers	No	
Inclusion Criteria:	Cigna customers: For each index patient in a particular cohort, Investigators randomly identified up to four children of the same ages as the index patient but who do not have the specific SPI. The matching by age was as follows: in months if < 3 years; and in years if age > or = 3 years. Cigna then identified all family members.	Similar	Cases and controls were matched if age was within 1 month.
Exclusion Criteria:	None	Yes	Cases and controls in the prematurity and cardiac cohorts were excluded if they had been the products of multiple birth (i.e. twins). A few cases were excluded because they matched with a control family with no medical coverage. A few families were excluded because no one in the case or control family had medical coverage after the index day.

© 2021 Feudtner C et al. *JAMA Network Open*.

eTable 3: Demographic characteristic of case and control children by cohort

Cohort	Characteristics		Cases	Controls	P value
**Prematurity**			N =1,176	N = 3,571	
			n (%)	n (%)	
	Age		Birth	Birth	
	Gender	Female	557 (47.4)	1,729 (48.4)	.53
		Male	619 (52.6)	1,842 (51.6)	
	Race/Ethnicity	Asian	54 (4.6)	207 (5.8)	<.001
		Black	114 (9.7)	219 (6.1)	
		Hispanic	108 (9.2)	299 (8.4)	
		White	653 (55.5)	2,287 (64.0)	
		Missing	247 (21.0)	779 (16.5)	
	Family Structure	Family size, Median (IQR)	3.0 (3-4)	4.0 (3-4)	<.001
		Adults in family, Median (IQR)	2.0 (1-2)	2.0 (2-2)	<.001
		Children in family, Median (IQR)	1.0 (1-2)	2.0 (1-2))	<.001
**Cardiac**			N = 911	N = 2,792	
			n (%)	n (%)	
	Age		Birth	Birth	
	Gender	Female	399 (43.8)	1,375 (49.3)	.004
		Male	512 (56.2)	1,417 (50.8)	
	Race/Ethnicity	Asian	50 (5.5)	148 (5.3)	.37
		Black	73 (8.0)	184 (6.6)	
		Hispanic	83 (9.1)	219 (7.8)	
		White	569 (62.5)	1,793 (64.2)	
		Missing	136 (14.9)	448 (16.1)	
	Family Structure	Family size, Median (IQR)	4.0 (3-4)	4.0 (3-4)	.95
		Adults in family, Median (IQR)	2.0 (2-2)	2.0 (2-2)	.60
		Children in family, Median (IQR)	2.0 (1-3)	2.0 (1-2)	.90
**Oncology**			N = 1,520	N = 3,777	
			n (%)	n (%)	
	Age	Mean (SD)	11.2 (5.0)	11.4 (5.0)	.36
	Gender	Female	673 (44.3)	1,937 (51.3)	<.001
		Male	847 (55.7)	1,840 (48.7)	
	Race/Ethnicity	Asian	78 (5.1)	204 (5.4)	.051
		Black	64 (4.2)	225 (6.0)	
		Hispanic	151 (9.9)	368 (9.7)	
		White	1,067 (70.2)	2,533 (67.1)	
		Missing	160 (10.5)	447 (11.8)	
	Family Structure	Family size, Median (IQR)	4.0 (4-5)	4.0 (3-5)	<.001
		Adults in family, Median (IQR)	2.0 (2-2)	2.0 (2-2)	<.001
		Children in family, Median (IQR)	2.0 (2-3)	2.0 (2-3)	<.001
**Neurologic**			N = 3,302	N = 8,479	
			n (%)	n (%)	
	Age	Mean (SD)	10.3 (5.4)	10.4 (5.4)	.872
	Gender	Female	1,385 (41.9)	4,179 (49.3)	<.001
		Male	1,917 (56.1)	4,300 (50.7)	
	Race/Ethnicity	Asian	150 (4.5)	466 (5.5)	.01
		Black	204 (6.2)	515 (6.1)	
		Hispanic	383 (11.6)	837 (9.9)	
		White	2,241 (67.9)	5,750 (67.8)	
		Missing	324 (9.8)	1907 (12.6)	
	Family Structure	Family size, Median (IQR)	4.0 (4-5)	4 (3-5)	<.001
		Adults in family, Median (IQR)	2.0 (2-2)	2.0 (2-2)	<.001
		Children in family, Median (IQR)	2.0 (2-3)	2.0 (2-3)	<.001

© 2021 Feudtner C et al. *JAMA Network Open*.

eTable 4: Comparison of mothers and fathers regarding healthcare encounters, diagnoses, and prescriptions

			Mothers			Fathers		
	Outcomes		Cases M(SD)	Controls M (SD)	IRR (95% CI)	Cases M (SD)	Controls M (SD)	IRR (95% CI)
**Encounters**								
	Premature	Hospitalizations	0.2 (0.5)	0.2 (0.5)	0.90 (0.76 to 1.08)	0.02 (0.2)	0.03 (0.2)	0.74 (0.44 to 1.25)
		ED Visits	0.2 (0.8)	0.1 (0.6)	*1.74 (1.36 to 2.22)	0.2 (0.6)	0.2 (0.6)	*1.37 (1.10 to 1.69)
		Urgent Care	0.2 (0.8)	0.2 (0.7)	1.08 (0.82 to 1.42)	0.2 (0.7)	0.2 (0.7)	1.16 (0.92 to 1.46)
	Cardiac	Hospitalizations	0.2 (0.5)	0.2 (0.4)	1.21 (0.99 to 1.47)	0.03 (0.2)	0.03 (0.3)	0.79 (0.40 to 1.58)
		ED Visits	0.2 (1.0)	0.2 (0.6)	1.31 (0.92 to 1.87)	0.2 (0.6)	0.2 (0.6)	1.06 (0.81 to 1.38)
		Urgent Care	0.2 (0.9)	0.2 (0.7)	1.25 (0.90 to 1.72)	0.2 (0.8)	0.2 (0.7)	1.13 (0.88 to 1.46)
	Oncology	Hospitalizations	0.09 (0.4)	0.1 (0.4)	0.96 (0.74 to 1.24)	0.06 (0.4)	0.06 (0.4)	1.11 (0.74 to 1.65)
		ED Visits	0.3 (0.8)	0.2 (0.9)	1.13 (0.92 to 1.40)	0.2 (0.8)	0.12 (0.6)	*1.27 (1.05 to 1.54)
		Urgent Care	0.3 (0.8)	0.2 (0.8)	1.13 (0.92 to 1.39)	0.2 (0.7)	0.2 (0.7)	1.11 (0.90 to 1.37)
	Neurologic	Hospitalizations	0.1 (0.4)	0.1 (0.4)	*1.28 (1.08 to 1.53)	0.1 (0.4)	0.04 (0.3)	1.17 (0.85 to 1.61)
		ED Visits	0.3 (1.1)	0.2 (1.0)	*1.19 (1.01 to 1.40)	0.2 (0.8)	0.2 (0.6)	1.16 (0.99 to 1.35)
		Urgent Care	0.3 (0.9)	0.3 (0.8)	1.14 (0.99 to 1.30)	0.2 (0.7)	0.2 (0.6)	*1.18 (1.02 to 1.38)
**Diagnoses**								
	Premature	Mental health	3.4 (11.2)	2.5 (10.2)	*1.28 (1.01 to 1.70)	1.9 (8.8)	2.0 (13.4)	0.99 (0.68 to 1.43)
		Physical health	42.3 (115.6)	26.7 (48.0)	*1.48 (1.30 to 1.69)	19.3 (37.4)	17.5 (38.5)	1.03 (0.88 to 1.20)
		Trauma	1.2 (6.3)	1.2 (7.5)	0.86 (0.60 to 1.23)	2.4 (13.6)	2.0 (10.8)	1.09 (0.74 to 1.62)
	Cardiac	Mental health	2.9 (11.1)	2.4 (10.2)	1.22 (0.86 to 1.73)	1.7 (6.4)	2.2 (19.8)	0.74 (0.49 to 1.11)
		Physical health	35.7 (110.5)	27.3 (48.1)	1.12 (0.96 to 1.29)	18.9 (35.4)	20.0 (46.1)	0.91 (0.77 to 1.08)
		Trauma	1.2 (8.2)	1.4 (8.1)	0.86 (0.59 to 1.24)	2.0 (9.6)	1.9 (9.3)	1.13 (0.73 to 1.75)
	Oncology	Mental health	5.0 (27.6)	2.6 (10.0)	*1.91 (1.44 to 2.52)	3.3 (15.5)	2.0 (12.1)	*1.66 (1.24 to 2.22)
		Physical health	40.6 (70.9)	33.5 (53.1)	*1.32 (1.17 to 1.48)	31.4 (76.1)	26.2 (58.7)	*1.34 (1.17 to 1.53)
		Trauma	2.4 (11.4)	2.2 (13.0)	1.10 (0.79 to 1.53)	2.5 (12.3)	2.1 (11.5)	1.22 (0.90 to 1.67)
	Neurologic	Mental health	5.0 (22.6)	2.8 (11.7)	*1.85 (1.54 to 2.23)	2.4 (10.0)	1.7 (8.0)	*1.37 (1.13 to 1.67)
		Physical health	45.8 (95.5)	31.8 (56.6)	*1.49 (1.37 to 1.62)	32.4 (72.7)	24.4 (57.5)	*1.42 (1.28 to 1.57)
		Trauma	2.2 (9.2)	1.8 (9.4)	*1.38 (1.13 to 1.69)	5.0 (28.2)	1.9 (9.6)	*1.26 (1.03 to 1.55)
**Prescriptions**								
	Premature	Mental health	2.1 (7.2)	1.7 (5.7)	*1.49 (1.09 to 2.05)	1.1 (4.2)	1.1 (5.0)	0.96 (0.65 to 1.42)
		All other	15.0 (21.1)	10.2 (14.2)	*1.46 (1.31 to 1.64)	7.4 (16.1)	6.5 (12.6)	1.05 (0.88 to 1.27)
	Cardiac	Mental health	2.0 (5.5)	1.7 (5.4)	1.11 (0.81 to 1.51)	1.5 (6.2)	1.2 (6.1)	1.16 (0.76 to 1.78)
		All other	11.4 (15.3)	10.0 (13.4)	*1.16 (1.01 to 1.32)	7.8 (15.6)	7.4 (15.2)	0.93 (0.77 to 1.12)

© 2021 Feudtner C et al. *JAMA Network Open*.

	Oncology	Mental health	3.6 (9.5)	2.3 (6.6)	*1.68 (1.30 to 2.17)	2.1 (7.1)	1.2 (4.2)	*1.72 (1.26 to 2.35)
		All other	14.1 (21.3)	11.5 (19.2)	*1.42 (1.25 to 1.62)	12.3 (21.3)	9.6 (17.4)	*1.55 (1.32 to 1.82)
	Neurologic	Mental health	3.9 (9.9)	2.3 (6.5)	*1.71 (1.46 to 1.99)	2.0 (7.0)	1.2 (4.6)	*1.69 (1.34 to 2.14)
		All other	16.4 (25.1)	11.5 (17.9)	*1.48 (1.36 to 1.61)	12.6 (22.9)	9.1 (16.2)	*1.49 (1.34 to 1.65)

Note. All negative binomial regression models adjusted for age of parent and duration of observation following the index date; * = p <.05.

© 2021 Feudtner C et al. *JAMA Network Open*.

eTable 5: Comparison of sisters and brothers regarding healthcare encounters, diagnoses, and prescriptions

			Sisters			Brothers		
	Outcomes		Cases M (SD)	Controls M (SD)	IRR (95% CI)	Cases M (SD)	Controls M (SD)	IRR (95% CI)
**Encounters**								
	Premature	Hospitalizations	0.02 (0.2)	0.03 (0.3)	0.44 (0.14 to 1.46)	0.04 (0.5)	0.02 (0.3)	2.61 (0.58 to 11.85)
		ED Visits	0.2 (0.8)	0.2 (0.6)	0.95 (0.63 to 1.42)	0.3 (0.7)	0.2 (0.6)	*1.35 (1.01 to 1.79)
		Urgent Care	0.2 (0.8)	0.2 (0.7)	1.33 (0.87 to 2.04)	0.2 (0.6)	0.2 (0.7)	1.15 (0.76 to 1.74)
	Cardiac	Hospitalizations	0.02 (0.1)	0.03 (0.4)	0.79 (0.33 to 1.88)	0.03 (0.4)	0.05 (0.8)	1.24 (0.35 to 4.35)
		ED Visits	0.2 (0.5)	0.2 (0.8)	0.82 (0.59 to 1.14)	0.3 (0.7)	0.2 (0.7)	1.22 (0.89 to 1.68)
		Urgent Care	0.2 (0.6)	0.2 (0.7)	0.93 (0.61 to 1.42)	0.1 (0.7)	0.2 (0.6)	0.70 (0.42 to 1.18)
	Oncology	Hospitalizations	0.05 (0.6)	0.04 (0.3)	1.70 (0.82 to 3.50)	0.02 (0.3)	0.03 (0.2)	0.95 (0.51 to 1.80)
		ED Visits	0.3 (1.0)	0.2 (0.9)	1.17 (0.90 to 1.53)	0.2 (0.7)	0.2 (0.6)	1.22 (0.96 to 1.52)
		Urgent Care	0.3 (0.8)	0.3 (0.8)	0.97 (0.75 to 1.43)	0.2 (0.7)	0.2 (0.7)	1.16 (0.91 to 1.47)
	Neurologic	Hospitalizations	0.04 (0.4)	0.0 (0.4)	1.36 (0.85 to 2.18)	0.02 (0.3)	0.03 (0.4)	0.88 (0.51 to 1.54)
		ED Visits	0.3 (1.0)	0.2 (1.4)	*1.51 (1.27 to 1.80)	0.3 (0.7)	0.2 (0.7)	*1.23 (1.06 to 1.43)
		Urgent Care	0.3 (0.9)	0.2 (0.8)	*1.29 (1.09 to 1.53)	0.2 (0.7)	0.2 (0.7)	*1.24 (1.04 to 1.49)
**Diagnoses**								
	Premature	Mental health	1.7 (7.6)	2.7 (51.8)	1.17 (0.65 to 2.11)	2.5 (16.9)	3.7 (35.8)	*0.53 (0.29 to 0.98)
		Physical health	11.9 (33.4)	14.1 (64.6)	0.83 (0.65 to 1.07)	15.5 (76.2)	11.1 (32.4)	1.41 (0.89 to 2.24)
		Trauma	1.0 (4.2)	1.1 (4.7)	0.93 (0.59 to 1.48)	1.7 (5.9)	1.3 (4.5)	1.09 (0.72 to 1.63)
	Cardiac	Mental health	1.0 (4.4)	1.6 (11.8)	0.73 (0.40 to 1.33)	5.2 (44.3)	3.9 (37.1)	1.52 (0.63 to 3.66)
		Physical health	12.1 (16.7)	15.0 (65.1)	0.89 (0.72 to 1.10)	12.0 (25.2)	13.6 (59.4)	0.86 (0.65 to 1.13)
		Trauma	1.1 (3.8)	1.3 (5.7)	0.89 (0.59 to 1.32)	1.7 (6.6)	1.3 (4.3)	1.33 (0.89 to 1.99)
	Oncology	Mental health	3.6 (17.6)	2.7 (14.2)	1.17 (0.77 to 1.77)	5.6 (42.6)	2.7 (16.0)	*1.99 (1.30 to 3.04)
		Physical health	17.2 (48.0)	13.2 (30.9)	*1.37 (1.14 to 1.65)	12.8 (28.8)	10.8 (38.3)	*1.30 (1.07 to 1.59)
		Trauma	2.0 (7.2)	1.6 (6.5)	1.33 (0.97 to 1.81)	2.3 (7.0)	2.4 (10.8)	1.20 (0.93 to 1.56)
	Neurologic	Mental health	3.9 (16.6)	2.2 (12.6)	*2.18 (1.68 to 2.83)	6.5 (81.0)	3.2 (29.5)	*1.91 (1.13 to 3.23)
		Physical health	21.1 (75.7)	13.2 (53.4)	*1.82 (1.55 to 2.14)	15.0 (42.5)	9.8 (34.9)	*1.60 (1.36 to 1.89)
		Trauma	2.0 (6.9)	1.6 (8.3)	*1.43 (1.17 to 1.75)	2.2 (7.1)	2.0 (11.0)	1.15 (0.95 to 1.40)
**Prescriptions**								
	Premature	Mental health	0.2 (1.6)	0.2 (2.8)	2.04 (0623 to 6.72)	0.1 (0.9)	0.3 (2.8)	*0.34 (0.12 to 0.95)
		All other	3.5 (8.1)	3.9 (8.0)	0.91 (0.69 to 1.19)	4.4 (8.3)	4.1 (8.5)	1.08 (0.83 to 1.42)
	Cardiac	Mental health	0.5 (3.5)	0.2 (2.4)	5.86 (0.77 to 44.28)	0.4 (3.5)	0.1 (1.3)	*5.21 (1.09 to 25.00)
		All other	4.7 (7.2)	3.5 (5.6)	*1.31 (1.04 to 1.66)	4.8 (10.0)	4.0 (12.9)	1.30 (0.90 to 1.87)

© 2021 Feudtner C et al. *JAMA Network Open*.

	Oncology	Mental health	0.8 (3.6)	0.5 (2.3)	*2.38 (1.35 to 4.19)	0.6 (4.0)	0.5 (2.7)	1.68 (0.77 to 3.70)
		All other	7.1 (12.5)	4.5 (7.3)	*1.97 (1.63 to 2.39)	5.1 (9.9)	3.2 (7.4)	*1.95 (1.61 to 2.35)
	Neurologic	Mental health	1.2 (6.3)	0.6 (3.1)	*2.09 (1.26 to 3.46)	0.8 (4.7)	0.3 (2.3)	*3.39 (1.97 to 5.83)
		All other	7.3 (13.3)	5.1 (11.0)	*1.75 (1.51 to 2.02)	5.6 (11.3)	3.3 (7.1)	*1.75 (1.50 to 2.03)

Note. All negative binomial regression models adjusted for age of sibling and duration of observation following the index date; * = p <0.05.

© 2021 Feudtner C et al. *JAMA Network Open*.

eTable 6: Comparison of bereaved mothers and fathers regarding mental, physical, and trauma healthcare diagnoses.

			Mothers			Fathers		
	Outcomes		Cases M (SD)	Controls M(SD)	IRR (95% CI)	Cases M (SD)	Controls M (SD)	IRR (95% CI)
**Bereaved**								
	Premature	Mental Health	4.7 (15.0)	2.5 (10.2)	*1.72 (1.06 to 2.82)	1.9 (8.1)	2.0 (13.4)	1.15 (0.44 to 3.02)
		Physical Health	40.0 (85.2)	26.7 (48.0)	*1.51 (1.10 to 2.06)	19.9 (40.8)	17.5 (38.5)	1.10 (0.78 to 1.57)
		Trauma	2.3 (12.3)	1.2 (7.5)	2.08 (0.85 to 5.09)	3.6 (20.9)	2.0 (10.8)	2.35 (0.67 to 8.16)
	Cardiac	Mental Health	4.7 (11.7)	2.4 (10.2)	*1.97 (1.07 to 3.62)	3.6 (6.9)	2.2 (19.8)	1.42 (0.70 to 2.86)
		Physical Health	36.4 (57.9)	27.3 (48.1)	1.28 (0.94 to 1.73)	20.4 (32.1)	20.0 (46.1)	0.99 (0.60 to 1.62)
		Trauma	1.7 (9.2)	1.4 (8.1)	0.89 (0.34 to 2.29)	0.5 (1.6)	1.9 (9.3)	*0.23 (0.09 to 0.61)
	Oncology	Mental Health	8.0 (20.4)	2.6 (10.0)	*3.48 (2.21 to 5.49)	6.6 (33.2)	2.0 (12.1)	*2.78 (1.18 to 6.54)
		Physical Health	36.4 (57.8)	33.5 (53.1)	1.02 (0.77 to 1.33)	32.4 (61.7)	26.2 (58.7)	*2.25 (1.29 to 3.91)
		Trauma	2.7 (10.7)	2.2 (13.0)	1.21 (0.61 to 2.43)	2.4 (8.4)	2.1 (11.5)	1.02 (0.54 to 1.93)
	Neurologic	Mental Health	8.4 (25.7)	2.8 (11.7)	*3.06 (1.86 to 5.03)	3.6 (10.9)	1.7 (8.0)	*1.97 (1.21 to 3.19)
		Physical Health	40.8 (56.9)	31.8 (56.6)	*1.40 (1.06 to 1.77)	51.2 (163.9)	24.4 (57.5)	*2.35 (1.32 to 4.20)
		Trauma	2.3 (9.7)	1.8 (9.4)	1.52 (0.79 to 2.91)	5.0 (28.2)	1.9 (9.5)	*2.40 (1.03 to 5.59)
